# First Detection of *Chlamydia trachomatis '*Swedish' Variant (nvCT) in a Russian Couple with Infertility

**DOI:** 10.2174/1874285801812010343

**Published:** 2018-10-18

**Authors:** Valentina Feodorova, Edgar Sultanakhmedov, Yury Saltykov, Sergey Zaitsev, Sergey Utz, Michael Corbel, Charlotte Gaydos, Thomas Quinn, Vladimir Motin

**Affiliations:** 1 Laboratory for Molecular Biology and NanoBiotechnology, Federal Research Center for Virology and Microbiology, Branch in Saratov, Ap. 6, the 53^rd^ Strelkovoi Divisii Street, Saratov, 410028, Russia; 2 Department for Skin Diseases, Saratov State Medical University, 22, Proviantskaya Street, Saratov, 410028, Russia; 3 Department of Bacteriology, The National Institute for Biological Standards and Control (NIBSC), Potters Bar, EN6 3QG, UK; 4 Division of Infectious Diseases, Johns Hopkins University School of Medicine, 855 N. Wolfe Street, Rangos Bldg, Suite 530, Baltimore, MD 21205, USA; 5 Division of Intramural Research, National Institute of Allergy and Infectious Diseases, Baltimore, MD, 21205, USA; 6 Department of Pathology & Immunology, University of Texas Medical Branch, 301 University Boulevard, Galveston, TX 77555-0609, USA

**Keywords:** *Chlamydia trachomatis*, Novel Swedish strain, Co-infection, Extra-genital site, Infertility, Detection methods

## Abstract

**Background:**

Chronic asymptomatic chlamydial genital infection caused by the wild-type of *Chlamydia trachomatis* (wtCT) is the most common bacterial infection causing human infertility. The novel ‘Swedish’ variant of *С.trachomatis* (nvCT) which contains a 377 bp deletion in a region that is specifically targeted in some nucleic acid amplification tests may impede diagnosis.

**Objective:**

The study aimed to investigate whether nvCT may be a possible cause of infertility in a couple undergoing *in vitro* fertilization (IVF).

**Method:**

Clinical specimens from both genital (urethra and cervix) and extra-genital sites (pharynx, conjunctive, blood) of a couple who experienced multiple unsuccessful attempts at pregnancy by natural fertilization and IVF procedures were analyzed before and after antibiotic therapy. Both partners had neither somatic nor endocrinal abnormality nor any clinically apparent genital manifestations of *Chlamydia* or other STIs.

**Results:**

Before antibiotic therapy all the samples of the Female Partner (FP) contained DNA of only the nvCT. After antibiotic therapy, additionally, DNA of wtCT of genovars E and D was detected in specimens from her conjunctiva and oropharynx. All samples of the Male Partner (MP) revealed co-infection of nvCT and wtCT. Identical SNP within the variable region 4 (VD4) of the *ompA* gene confirmed the identity of the wtCT strains found in both partners. The FP had a positive anti-chlamydial IgG titer. The sperm characteristics of the MP, motility (immotile spermatozoa was 51.1% versus 21.6%) and vitality (46% versus 68%) declined progressively, and the MP anti-chlamydial IgG titer was negative.

**Conclusion:**

Infertility in this couple may have been caused by chronic asymptomatic and persistent nvCT-associated infection that was complicated by re-infection later with wtCT. This study illustrates the importance of including detection methods for nvCT strains in the investigation of infertility cases.

## 1 INTRODUCTION

*Chlamydia trachomatis* is currently one of the most prevalent sexually transmitted pathogens worldwide and a major cause of infertility affecting 10-15% of all couples worldwide [1-4]. The essential role of the wild type of *С.trachomatis* (wtCT) in human infertility is well-established [2, 4]. A number of animal studies also present clear evidence that human strains of wtCT are capable of causing infertility [5, 6]. This wtCT-associated infection can result in alteration of semen parameters, induction of apoptosis in spermatozoa, and decrease in the reproductive performance of fertile male mice [5]. The relevance of this observation was confirmed in recent studies where *in-vitro* co-incubation of human sperm with wtCT (serovar E) caused a significant decline in the number of live and motile spermatozoa, stimulation of an apoptosis-like response in spermatozoa and premature sperm death [2, 7, 8]. In the female, wtCT is a major cause of pelvic inflammatory disease, leading to chronic abdominal pain, ectopic pregnancy and tubal factor infertility [1, 4]. The wtCT genital chlamydial infections are frequently chronic and asymptomatic [2, 4]. If undiagnosed, wtCT can produce poor outcomes with respect to infertility problems including failure of both natural and assisted conception techniques, such as *in vitro* fertilization (IVF) [4, 9]. Unfortunately, the actual contribution of chlamydial infection caused by the novel ‘Swedish’ variant of *С.trachomatis* (nvCT) to couple infertility remains poorly investigated. This chlamydial variant, which carries a 377 bp deletion within the cryptic plasmid that has caused false-negative responses in routine nucleic acid amplification tests (NAATs) [10, 11] is a novel challenge for laboratory diagnosis of chlamydial infection worldwide. At first, this type of strain was difficult to detect with commercial NAATs, since the deletion was located within the *orf1* of the plasmid targeted by diagnostics. For the past ten years, the nvCT strains have been intensively studied with respect to their phenotypic and biological characteristics. Based on whole genome sequencing, molecular epidemiology and *in vitro* research data, characteristics of the nvCT were found to be similar to those of wtCT [2, 12-15]. Nevertheless, the nvCT strains exceeded the wild type strains in their ability to disseminate rapidly in the human population that was attributed to the problems of initial difficulties in detection of such variants [12]. However, little is known about the contribution of the nvCT to the development of chronic persistent chlamydial genital infection, infertility, clinical manifestations, extra-genital forms of the disease, and their ability to compete with the wtCT during mixed infection. Also, it is important to determine whether the nvCT can induce chlamydial infection as a sole etiologic factor, or if it requires a co-infection with the wtCT variant. This knowledge should improve routine laboratory diagnostics and treatment of genital infection caused by both wtCT and nvCT.

In this study, we report an investigation of chronic asymptomatic chlamydial genital infection involving extensive molecular testing in a sub-fertile couple that underwent multiple unsuccessful attempts at pregnancy by both natural fertilization and IVF procedures.

## 2 MATERIALS AND METHODS

### 2.1 Brief Personal and Clinical Characteristics of Patients

Female and male partners of 25 and 28 years old, respectively, comprised a heterosexual married couple from Saratov city. During the past six years, they had frequent sexual contacts to attempt pregnancy. Moreover, this couple had been engaged in receptive oral and genital, but not anal intercourse. Each individual had regular annual physical and laboratory examinations by a gynecologist or andrologist, an endocrinologist and a family physician. Both partners had neither somatic nor endocrinal abnormality nor symptoms, nor any clinically apparent genital manifestations of *Chlamydia* or other Sexually Transmitted Infections (STIs). Nevertheless, they demonstrated marked symptoms of bacterial conjunctivitis, such as unilateral hyperemia and mucopurulent discharge that were observed for the last 6 months. During the last three years, the patients underwent several unsuccessful attempts at IVF. The female partner disclaimed any sexual contacts outside legal marriage. However, the male partner reported frequent and irregular relationships with at least 3 to 5 female partners annually. Both partners denied any pernicious habits or addiction to alcohol, as well as animal or homosexual contacts. Both of them declared that they had never traveled to Sweden or other EU countries.

### 2.2 Clinical Samples

Clinical specimens were taken in October 2013, from both genital (urethra & cervix) and extra-genital (oropharynx, conjunctive, blood) sites of each individual one year after a single course of antibiotic therapy. Clinical samples from similar sites of both partners were also obtained in October, 2012 prior to initiation of antibiotic therapy according to recommendations of the European STD Guidelines, and CDC STDs Treatment Guidelines [16, 17]. All procedures performed in studies involving human participants were in accordance with the ethical standards of the 1964 Helsinki declaration and its later amendments or comparable ethical standards. The research protocol was approved by the Human Bioethics Committee of the Saratov Scientific and Research Veterinary Institute No. IRB00008288 (http://ohrp.cit.nih.gov/search/IrbDtl.aspx), and both participants provided written informed consent.

### 2.3 Semen Analysis

Semen analysis was performed on the male partner according to the procedures outlined by the World Health Organization (WHO) [18], twice in October 2012, and three times in August 2014 by means of an automated sperm analyzer SQA-V (MES, Austria-Israel).

### 2.4 Detection of *C.trachomatis* by Monoclonal Antibodies

Direct Immunofluorescent Test (DIFT) with a mixture of chlamydia monoclonal antibodies (MAbs) to synthetic serogroup-specific MOMP-associated epitopes of 17 *С.trachomatis* serovars A-L (NiarMedic Plus, Moscow, Russia) [19] was used to test for chlamydial Elementary Bodies (EBs) in individual clinical samples.

### 2.5 Detection of *C.trachomatis* Plasmid and Chromosomal DNA by PCR and Genotyping

Total DNA was isolated from clinical samples including blood specimens using DNeasy Blood and Tissue Kit (Qiagen, Hilden, Germany). Each DNA sample was tested by: (i) End-point PCR with conventional and fluorescent product detection using commercial kits developed by the Central Research Institute of Epidemiology, Moscow, Russia (AmpiSens-Eph and AmpiSens-FL, respectively) to reveal the plasmid DNA of the typical *C. trachomatis* strains (wtCT) in the clinical material [20]; (ii) PCR with two pairs of primers, such as *orf2*_F/*orf2*_R and *orf8*_F/*orf8*_R (Table **1**), designed by us to recognize genes *orf2* and *orf8* of *C. trachomatis* plasmid (PCR-orf2 and PCR-orf8, respectively).

We used plasmid pBour of *C. trachomatis* strain E/Bour (GeneBank accession no. HE603212) to obtain sequences of both *orf2* and *orf8* genes. These primers amplified fragments of 150 bp and 145 bp for *orf2* and *orf8*, respectively. The reaction was done in a 30 µL volume with 10 pmol of each primer, and 1 µL of DNA isolated from the clinical sample. The conditions of amplification were as follows: 94° C for 2 min, 15 cycles of 94° C for 30 sec, 61° C for 20 sec, and 72° C for 30 sec followed by 45 cycles of 94° C for 30 sec, 58° C for 20 sec, and 72° C for 45 sec with the final extension at 72° C for 10 min; (iii) PCR with the pair of primers developed by Catsburg *et al.* [21] to identify the presence of DNA of novel Swedish strains of *C. trachomatis* (nvCT) – PCR-nvCT, respectively. The forward primer swCT_serE_F was used in the original design. The reverse primer swCT_serE_R was modified by us in a single nucleotide to fit better the sequence of genovar E (Table **1**). These primers amplified a product with a size of 474 bp and 97 bp in the presence of wtCT and nvCT templates, respectively. The DNA of genovar E of *C. trachomatis* strain E/Bour (#VR-348BD, ATCC) was used as a control for each experiment. The reaction was done in 30 µL with 10 pmol of each primer, and 1 µL of DNA isolated from the clinical sample. The conditions of amplification were the following: 94° C for 2 min, 35 cycles of 94° C for 30 sec, 57° C for 30 sec, and 72° C for 45 sec followed by the final extension at 72° C for 10 min. Then, the fragments of amplification were purified from gel by using the QIAquick Gel Extraction Kit (Qiagen), and sequenced commercially by the EVROGEN Company (Moscow, Russia).

The individual *C. trachomatis*-positive DNA samples were genotyped by sequencing of the variable domain 2 (VD2) region of *ompA* as previously described by Quint *et al.* [22]. This system of genotyping included three forward and four reverse primers that in a multiplex format could amplify the VD2 region of all known *C. trachmatis* genovariants generating amplicons of 157 to 160 bp. The fragments of amplification were extracted from gel and sequenced. To determine the genovar, the sequence reads from the forward and reverse primers were searched in the GenBank for homology by the BLAST program.

The *ompA* gene was amplified with the use of primers F11 and B11, which produced the 1156 bp fragment containing all four variable regions of the gene [23]. To sequence the amplicons, additional primers complementary to the internal part of the *ompA* gene were designed (MOMP-E_F1.seq; MOMP-E_F3.seq; MOMP-E_F5.seq; MOMP-E_R2.seq; MOMP-E_R4.seq; MOMP-E_R6.seq, Table **1**, Fig. S1) allowing reading of the fragment sequence at least twice from the direct and reverse chains. The sequencing was done commercially by the EVROGEN Company. The consensus sequence of the *ompA* derived from the DNA of clinical sample was compared with the reference sequences of the strains of genovar E, such as E/Bour (HE601870), E/IU-TC0755ut (FJ261948), and E/H38 (AF265238), as well as strains of the genovar D, such as D/SotonD1 (HE601798), D/SotonD5 (HE601799) as reported by Harris *et al.* [13]. The nucleotide sequence alignment was done using the MultAlin (http://multalin.toulouse.inra.fr/multalin/multalin.html program).

All representative genovar sequences reported in this research were deposited in GenBank (accession numbers MF288583-MF288585).

### 2.6 Detection of STIs DNA by PCR

Each DNA sample was also tested by real-time multiplex commercial kit developed by the Vector-Best (Novosibirsk, Russia) to reveal DNA of *Ureaplasma* spp., *Mycoplasma hominis*, *Mycoplasma genitalium*, *Trichomonas vaginalis*, *Neisseria gonorrhoeae*, *Candida albicans*, *Gardnerella vaginalis*, cytomegalovirus, herpes simplex virus (HSV) types 1/2 and human papillomavirus (HPV) types 16 and 18 [20].

### 2.7 Determination of Chlamydial IgG titres

*C.trachomatis* immunoglobulin G (IgG) antibody titres (CAT) of the patients were tested with an ELISA kit (“VECTOR-BEST” Company, Novosibirsk-117, Russia) according to the manufacturer’s instructions. Results were considered to be positive at ELISA titers ≥1:32.

## 3 RESULTS

### 3.1 Detection of nvCT in the Genital Samples of both Female and Male Patients

The initial screening in October 2012 prior to the planned IVF procedure revealed the lack of *С.trachomatis* DNA in the genital specimens (cervix and urethra) of both partners using AmpliSens *Chlamydia trachomatis*-Eph and -FL kits (AmpiSens-Eph and AmpiSens-FL). Nevertheless, specific chlamydial antigens were identified in these clinical specimens by DIFT (data not shown).

Further we tested the samples for the presence of chlamydial DNA in PCR by using primers targeting different parts of the *C. trachomatis* cryptic plasmid (*orf2* and *orf8*, PCR-orf2 and PCR-orf8), as well as primers swCT_serE_F/swCT_serE_R (Table **1**) flanking the area of deletion identified in the *orf1* of the Swedish strains (nvCT, PCR-nvCT, respectively). Although amplification with the primers recognizing both *orf2* and *orf8* provided negative results, specific DNA of the Swedish nvCT variant bearing a cryptic plasmid with a 377-bp deletion in the *orf1* was detected in the samples from the genital sites tested. The exact position of the 377-bp specific deletion within the *orf1* plasmid gene was confirmed by sequencing (data not shown). Additional examination of the female patient revealed scanty mucous vaginal discharge and moderate hyperemia in uterine and cervical mucosa in the absence of symptoms or any other clinical manifestations. After a single course of antibiotic therapy with doxycycline (Unidox Solutab®) 100 mg twice a day for seven days, and subsequent analysis of clinical specimens one year later (October 2013), her genital samples (urethra and cervix) were negative for wtCT DNA; however, the PCR analysis still revealed the presence of nvCT DNA in her cervix (Table **3**). The urethral specimen of the male partner was also initially (October, 2012) positive for nvCT alone (Table **2**). Later (October, 2013), in contrast to the results of the female patient testing, we detected chlamydial DNA with primers recognizing both wtCT and nvCT variants suggesting mixed infection. The presence of chlamydial EBs in clinical samples from genital sites of both partners was confirmed by DIFT (data not shown).

### 3.2 Presence of Extra-genital *C.trachomatis* Infection

During the first screening in October 2012, both AmpiSens-Eph and AmpiSens-FL kits failed to detect the wtCT DNA in extra-genital specimens of both partners. PCR-orf2 did not detect the presence of *Chlamydia*. Nevertheless, PCR-orf8 revealed *Chlamydia* in specimens derived from two out of three extra genital sites (conjunctiva and oropharynx). Moreover, PCR-nvCT produced positive signals with all three of their extra-genital samples, namely conjunctiva, oropharynx and blood (Table **2**). Testing the specimens obtained one year later showed that both male and female patients were positive for *Chlamydia* in all extra-genital sites. AmpiSens-Eph and AmpiSens-FL were less efficient, although both PCR-orf2 and PCR-orf8 produced positive reactions in most specimens. PCR-nvCT detected *Chlamydia* in all samples. Overall, both wtCT and nvCT were detected indicating a mixed infection in extra-genital sites of this couple. Chlamydial EBs were found by DIFT in all clinical samples.

### 3.3 Co-infection with nvCT and wtCT Genovars and Marker SNPs

The *ompA* genotype was identified as genovar E in all clinical samples from genital sites and in each of the three extra-genital sites tested (Tables **2** and **3**). Only novel ‘Swedish’ variant (nvCT) of *С.trachomatis* genovar E was detected in the patients at the first screening (Table **2**). One year later, specific chlamydial DNA of genovar D was also found in extra-genital sites of both partners, namely in conjunctiva and oropharynx of the female partner and in oropharynx of her husband, indicating a mixed infection in these extra-genital sites (Table **3**). When we determined the nucleotide sequence of the *ompA* gene of these genovar D variants, we identified SNPs that are not present in the current version of the GenBank for this genotype. The SNP at position 971 was matched in the samples obtained from the oropharynx of both partners, suggesting the identity of the strains that infected both patients. This SNP displayed a C→A substitution (compared with D/SotonD5) that resulted in an alanine-to-aspartic acid residue change within the VD4 region of the MOMP. Moreover, two additional SNPs were found in the conjunctiva of the female patient, those at positions 991 and 1020 (Table **3**). The SNP at position 991 was also located within the VD4 region, and displayed an A→G substitution resulting in a threonine-to-alanine amino acid change. The SNP at position 1020 was mapped at the second amino acid after the end of the VD4 region. The A→C substitution did not change the amino acid residue. A single SNP for the genovar E was identified at position 997 in the blood sample of the male patient. This SNP displayed a G→A substitution (compared with E/Bour) that resulted in an alanine-to-threonine amino acid change within the VD4 region of the MOMP. The *ompA* sequence found in this clinical specimen was identical with that existing in the strains E/IU-TC0755ut and E/H38 found in the GenBank. Importantly, since AmpiSens-Eph and AmpiSens-FL recognized the typical strains of *С.trachomatis* (wtCT), such as E/Bour, PCR-nvCT detected the ‘Swedish’ variant with the 377-bp deletion in the *orf1* of the cryptic plasmid. The *ompA* sequence analysis revealed the strain with the SNP at position 997, and there were at least three different strains of the genovar E present in the clinical specimens of our two patients.

### 3.4 Chlamydial IgG Titres in Sera of the Patients

The female partner demonstrated a CAT IgG titre of 1:160 at both initial screen and on re-examination one year later. No CAT IgG was registered in the male partner during this study.

### 3.5 Decreased Sperm Motility and Vitality in the Male Partner

The characteristics of the sperm in samples obtained in 2014 in comparison with those taken in 2012: progressive were, 29.8% versus 45% (the lower reference limit is 32%); non-progressive, 19.1% versus 23.4%; immotile, 51.1% versus 21.6%; vitality 46% versus 68% (the lower reference limit is 58%); pH 7.9 versus 7.6 (normal range is 7.2 – 7.8). Other major sperm parameters, such as total fluid volume, and spermatozoa concentration, were 4.5 ml (the lower reference limit is ≥ 1,5 ml) and 76.5 × 10^6^/ml (the lower reference limit is 15 × 10^6^ spermatozoa per ml), respectively, were maintained at the normal level. Overall, the sperm quality of the male partner collected in 2014 had declined after two years of chlamydial infection.

## 4 DISCUSSION

In this study, we carefully investigated both asymptomatic partners in a couple with infertility and recurrent chlamydial genital infection. At the beginning of this investigation, the partners demonstrated neither clinical nor laboratory manifestations of infertility as indicated also by male initial semen analysis prior to their first visit to the IVF Center. Additionally, their genital specimens, including male first void urine, considered as the most acceptable sample for detection of *С.trachomatis* in men [2] was negative for *С.trachomatis* when tested by commercial Russian kits AmpliSens-Eph and –FL that could consistently detect plasmid DNA of the typical wtCT strains [20], as well as tests for other STIs. Nevertheless, the female partner was seropositive for chlamydial antigens. Therefore, both partners received adequate treatment (doxycycline, 100 mg twice a day during 7 days) [16, 17] to prevent possible reinfection of this couple; however, not all the other multiple sexual partners of the male patient underwent antibiotic treatment.

Because multiple attempts at pregnancy conducted by the IVF procedure were unsuccessful, specimens from both female and male patients were investigated in our Reference Chlamydial laboratory, since a strong association between asymptomatic chlamydial genital infection in men and unexplained infertility has been suggested in literature [3]. In contrast to the new CDC Recommendations of 2014 for laboratory screening for *Chlamydia* in patients with and without symptoms of infection [24], the Russian standard laboratory protocol does not require testing of rectal samples from patients who are not engaged in anal intercourse. Therefore rectal specimens were omitted from this study, and cervical and urethral samples were analyzed. Additionally, we tested conjunctival, pharyngeal and blood specimens, since *С.trachomatis* antibodies can be detected in peripheral blood of patients with chronic asymptomatic chlamydial infection [25] and dissemination of *С.trachomatis* from the genital site to other intra-host sites has been documented in a murine model [26]. Moreover, both partners exhibited definite clinical symptoms of unilateral chronic bacterial conjunctivitis that likely were clinical manifestations of adult chlamydial conjunctivitis.

Surprisingly, the nvCT was identified in 100% of clinical specimens from either genital or extra-genital sites of both partners solely or as co-infection with wtCT in single sites (Tables **2**, **3**). These data provided a strong indirect evidence of possible association between the nvCT- infection and sub-fertility in this couple who were free from other STIs that are considered as potential bacterial species causing infertility including *N. gonorrhoeae*, *U. urealyticum*, and *M. genitalium* [27], as documented by conventional and real-time multiplex PCRs (data not shown). The screening for the relevant specific antibodies to these pathogens in sera of these patients also produced negative results.

In contrast to observations indicating the absence of significant differences in clinical manifestation between the nvCT and the wtCT strains [14, 15], we found no chlamydial genital symptoms in the male patient and only mild cervicitis in his female partner. Also contrary to the results of a high-risk city population attending a STIs clinic [15], the two individuals involved in the current study declared themselves perfectly healthy; the female patient did not have lower abdominal pain, pronounced vaginal discharge, frequent urination, post-coital bleeding, inter-menstrual bleeding, or any other complaints that were suggestive of a possible infection with *Chlamydia*. A complete absence of any symptoms of chlamydial genital infection in this couple caused by nvCT was distinct from the symptomatic infections observed for the wtCT strains by Bjartling *et al.* [15].

In fact, no major genetic difference was found between genomes of the nvCT and the wtCT strains suggesting that there is no alteration in biological fitness between two variants, which demonstrated high similarity with respect to epidemiological distribution and minimal differences in clinical signs in the vast majority of Chlamydia patients [28]. The transmission studies of the new variant of *C trachomatis* in Sweden conducted for the past ten years also showed that nvCT is clonal and genetically stable. Moreover, difficulties in detecting the nvCT variant did not affect the complication rates [29].

Retrospective analysis of the records of 2012 obtained from the IVF Clinic, such as hormonal status for the female patient, and semen analyses of the male partner [4, 18] excluded any endocrine disorder in the woman, but the results of semen analysis of her male partner were normal only initially in 2012. The same analysis conducted two years later registered a dramatic decrease in at least two main parameters, sperm motility and spermatozoa vitality, that had markedly deteriorated by about 1.5-fold compared with the initial test value, and were considered to be sub-normal as specified by the WHO [18]. Also a slight increase in ejaculate pH towards alkaline value was detected by this analysis. Although other sperm characteristics were in the normal range [18], the detected abnormalities in sperm could contribute to the failed results of the IVF procedure. These findings generally correlated with recent reports on the capability of human strains of wtCT to cause a significant decline in a number of vital and motile sperm in humans and mice, resulting in a marked decrease of its reproductive performance [5-8]. The development of these pathological changes found in the male patient took about 17 months.

These data indicated that nvCT may have a certain ability to infect both genital and extra-genital sites. Thus, including extra-genital sites in chlamydial diagnostic protocols can assist the efficient detection of this infection. The optimal detection of *С.trachomatis* in clinical specimens is possible only when appropriate targets for diagnostic assays are chosen [2]. A striking discrepancy in the ability of PCRs based on different targets used to detect *С.trachomatis* DNA in genital and extra-genital clinical specimens of our patients was observed (Tables **2**, **3**).

The important finding of our work was evidence of co-infection of the nvCT of genovar E and typical strains of wtCT of either E or D genovars in both patients during the second year of observation. The sequence analysis revealed the co-infection in most samples from conjunctiva and oropharynx (Table **3**). We did not identify any previous publications reporting co-infection with nvCT and wtCT variants, although our data suggest that such combination may occur more often than expected. The role of a sole nvCT or a mixed nvCT and wtCT infection in human infertility requires further study. Recently, a simultaneous association of two wtCT genovars (either D and E or F and K) was reported with regard to the evaluation of treatment efficacy, reinfection and drug resistance [30-32]. In our case, multiple sexual contacts of the male patient apart from his marriage partner are a likely explanation for the finding of different genovars of *С.trachomatis* in the couple’s specimens. Additionally, genovar E of the nvCT was first identified in this couple as a sole cause of *Chlamydia* infection, followed by two genovars of wtCT, D and E, identified during re-examination one year later in both partners (Table **3**). This reinfection from a promiscuous male partner nullified the effect of the antibiotic treatment, and may have increased the likelihood of failure of the IVF. This case is a good example of recorded history of sexual behavior and clear transmission of *С.trachomatis* strains of different genovars among sex partners [33]. The use of SNP analysis within the *ompA* gene allowed further identification of the strains infecting both partners. The SNP at position 997 in the blood sample of the male patient was detected first during the screening of urogenital samples for *С.trachomatis* in a Swedish clinic in 1999-2000 [34]. We also identified the unique SNP at position 971 in genovar D in the specimens of both patients, indicating a similar origin of these strains. The presence of other polymorphisms, such as unique SNPs at positions 991 and 1020 in serovar D, in the samples of each individual patient suggested even wider co-infection that took place in this clinical case.

## CONCLUSION

Finally, it can be concluded that the nvCT strain could be a cause of infertility in the male partner. Moreover, the deterioration in his semen quality appeared to begin when nvCT was the only strain detected, suggesting that it was a significant contributor. This illustrates the importance of including detection methods for nvCT strains in the investigation of infertility cases.

## Figures and Tables

**Table 1:** Primers used to amplify *C. trachomatis* DNA^a^.

**Primer**	**Sequence**	**Reference**
*orf8*_F	TCTTCTGCTTACAATGCTCTTGC	This study
*orf8*_R	CATCGACCTTGGTTTTTAAATCG	This study
*orf2*_F	AGCGAGTTACGAAGACAAAACCTC	This study
*orf2*_R	AGATTTGTTTCCAACAAGCTACCA	This study
swCT_serE_F	TCCGGATAGTGAATTATAGAGACTATTTAATC	[1]
swCT_serE_R	GGTGTTTGTACTAGAGGAATTACCTCTTC	This study
Fw1	TTCAATTTAGTTGGATTGTTTGG	[2]
Fw2	TCAACTTAGTTGGCTTATTCGG	[2]
Fw3	TCAATTTAGTGGGGTTATTCGG	[2]
Rv1	CACATTCCCAGAGAGCTGC	[2]
Rv2	CACATTCCCACAAAGCTGC	[2]
Rv3	CGGACTCCCACAAAGCTGC	[2]
Rv4	GCACTCCCACAAAGCTGC	[2]
F11	ACCACTTGGTGTGACGCTATCAG	[3]
B11	CGGAATTGTGCATTTACGTGAG	[3]
MOPM-E_F1.seq	CGGTATTAGTATTTGCCGCTTTG	This study
MOPM-E_F3.seq	CGACATATGCAGGATGCTGAG	This study
MOPM-E_F5.seq	TACCATGAGTGGCAAGCAAGTT	This study
MOPM-E_R2. seq	GAATACATCAAAGCGATCCCAAA	This study
MOPM-E_R4. seq	GATTGAGCGTATTGGAAAGAAGC	This study
MOPM-E_R6. seq	TGCTCGAGACCATTTAACTCCA	This study

^a^Primers for this study were designed by the Primer 3 program (http://bioinfo.ut.ee/primer3-0.4.0/primer3/.

**Table 2:** Detection of *Chlamydia* from genital and extra genital sites of the female and male partners in clinical specimens obtained in 2012.

**Clinical samples**	**Identification of *С. trachomatis* by PCR**											
	**Female partner**						**Male partner**					
	AmpiSens- Eph	AmpiSens- FL	PCR-nvCT ^a)^	PCR-*orf8*	PCR-*orf2*	Genovar^b)^	AmpiSens- Eph	AmpiSens- FL	PCR-nvCT ^a)^	PCR-*orf8*	PCR-*orf2*	Genovar^b)^
Conjunctiva	-	-	+	+	-	E	-	-	+	+	-	E
Oropharynx	-	-	+	+	-	E	-	-	+	-	-	E
Urethra & Cervix	-	-	+	-	-	E	-	-	+	+	-	E
Blood	-	-	+	-	-	E	-	-	+	-	-	E

^a)^ Presence of 377 bp deletion in *orf1* of the cryptic plasmid; ^b)^ *C. trachomatis* genovar was determined by sequencing of the VD2 region of the *ompA* gene.

**Table 3:** Detection of *Chlamydia* from genital and extra genital sites of the female and male partners in clinical specimens obtained in 2013.

**Clinical samples**	**Identification of *С. trachomatis* by PCR**											
	**Female partner**						**Male partner**					
	Ampi Sens-Eph	PCR-nvCT^a)^	PCR-*orf2*	PCR-*orf8*	Geno-var^b)^	SNP, position^c)^	Ampi Sens- Eph	PCR-nvCT^a)^	PCR-*orf2*	PCR-*orf8*	Geno-var^b)^	SNP, position^c)^
Conjunctiva	-	+	-	+	D E	991, 1020 None	-	+	-	+	E	None
Oropharynx	+	+	-	+	D E	971 None	-	+	+	-	D E	971 None
Urethra & Cervix	-	+	-	-	E	None	+	+	-	+	E	None
Blood	-	+	+	+	E	None	-	+	+	-	E	997

^a)^ Presence of 377 bp deletion in *orf1* of the cryptic plasmid; *^b)^ C. trachomatis* genovar was determined by sequencing of the VD2 region of the *ompA* gene; ^c)^ Single nucleotide polymorphism detected in the *ompA* gene: the SNP position compared to the genovars E and D, strains E/Bour and D/SotonD5, respectively.

## SUPPLEMENTARY MATERIAL

Supplementary material is available on the publishers Web site along with the published article.
