# Supplementary material for: First Detection of Chlamydia trachomatis 'Swedish' Variant (nvCT) in a Russian Couple with Infertility
Source: Open Microbiol J. 2018 Oct 18;12:343–52. doi: 10.2174/1874285801812010343 (PMC6198409; doi:10.2174/1874285801812010343)
Supplement: Supplementary file 1 [file TOMICROJ-12-343_SD1.pdf]

Send Orders for Reprints to reprints@benthamscience.ae

BENTHAM OPEN

CrossMark

The Open Microbiology Journal

Supplementary Material

Content list available at: www.benthamopen.com/TOMICROJ/

DOI: 10.2174/1874285801812010343, 2018, *12*, i

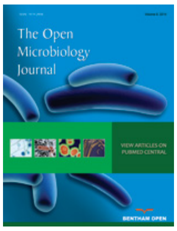

RESEARCH ARTICLE

First Detection of *Chlamydia trachomatis* 'Swedish' Variant (nvCT) in a Russian Couple with Infertility

Valentina Feodorova[1,*], Edgar Sultanakhmedov[2], Yury Saltykov[1], Sergey Zaitsev[1], Sergey Utz[2], Michael Corbel[3], Charlotte Gaydos[4], Thomas Quinn[4,5] and Vladimir Motin[6,*]

[1]*Laboratory for Molecular Biology and NanoBiotechnology, Federal Research Center for Virology and Microbiology, Branch in Saratov, Ap. 6, the 53rd Strelkovoi Divisii Street, Saratov, 410028, Russia*

[2]*Department for Skin Diseases, Saratov State Medical University, 22, Proviantskaya Street, Saratov, 410028, Russia*

[3]*Department of Bacteriology, The National Institute for Biological Standards and Control (NIBSC), Potters Bar, EN6 3QG, UK*

[4]*Division of Infectious Diseases, Johns Hopkins University School of Medicine, 855 N. Wolfe Street, Rangos Bldg, Suite 530, Baltimore, MD21205, USA*

[5]*Division of Intramural Research, National Institute of Allergy and Infectious Diseases, Baltimore, MD, 21205, USA*

[6]*Department of Pathology & Immunology, University of Texas Medical Branch, 301 University Boulevard, Galveston, TX 77555-0609, USA*

Received: July 14, 2018 Revised: September 19, 2018 Accepted: September 23, 2018

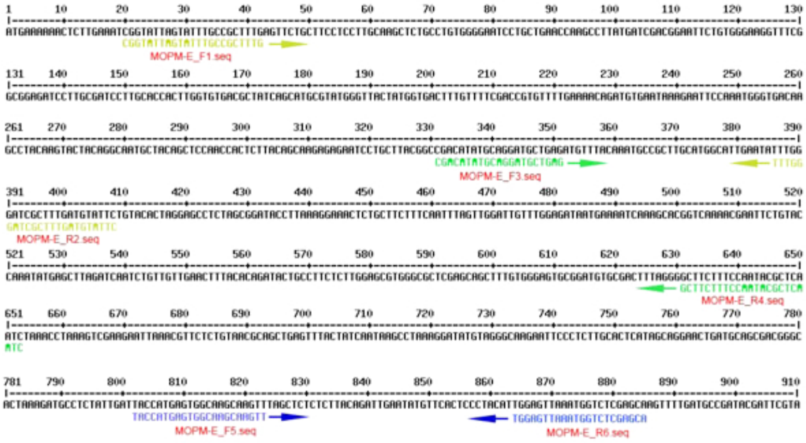

Supplemental Fig. (1). A schematic figure showing the position and sequence of six MOMP primer sets (MOMP-E_F1.seq; MOMP-E_F3.seq; MOMP-E_F5.seq; MOMP-E_R2.seq; MOMP-E_R4.seq; MOMP-E_R6.seq) targeting the *C. trachomatis ompA* gene region. Arrows indicate the direction for each of the primers.

© 2018 Feodorova *et al*.

This is an open access article distributed under the terms of the Creative Commons Attribution 4.0 International Public License (CC-BY 4.0), a copy of which is available at: (https://creativecommons.org/licenses/by/4.0/legalcode). This license permits unrestricted use, distribution, and reproduction in any medium, provided the original author and source are credited.
